# In ovo inoculation of duck embryos with different strains of *Bacillus cereus* to analyse their synergistic post‐hatch anti‐allergic potentialities

**DOI:** 10.1002/vms3.279

**Published:** 2020-05-04

**Authors:** Salauddin Al Azad, Khondoker Moazzem Hossain, Sana Mohammad Mahbubur Rahman, Mohammad Faysal Al Mazid, Pallob Barai, Mohammad Shamim Gazi

**Affiliations:** ^1^ Biotechnology and Genetic Engineering Discipline Life Science School Khulna University Khulna Bangladesh

**Keywords:** 16S rRNA gene sequencing, anti‐allergic potentialities, antimicrobial activity, *Bacillus cereus*, in ovo inoculation, probiotic properties

## Abstract

**Background:**

*Bacillus cereus* is a Gram‐positive, facultative anaerobic bacteria with few strains reported to be used as probiotics for animals and birds in recent times if the doses are formulated properly.

**Objectives:**

To analyse the synergistic anti‐allergic potentiality of different *Bacillus cereus* strains on experimental in ovo and in vitro duck model, as probiotic immune stimulant.

**Materials and methods:**

Different strains of *Bacillus cereus* from 29 isolates were identified through 16S rRNA gene sequencing from the milk samples of buffalo breeds of South Asia. The probiotic properties were tested in aspects of gram staining, catalase test, coagulase, test, bile salt tolerance, pH tolerance and phenol tolerance test. MIC_50_ and MIC_90_ levels were profiled using nine different antibiotics, and antimicrobial activity against eight different enteric pathogens was assessed*.* Finally, the test strains of *Bacillus cereus* (Colony Forming Unit [CFU] 30X10^11^) were combined‐infused at different concentrations in embryonated duck eggs to assess the post‐hatch anti‐allergic effects against histamine‐induced allergic reaction and their immunoglobulin E (IgE) level was tested.

**Results:**

Molecular identification confirmed the test strains as *B. cereus* HKS 1–1, *B. cereus* LOCK 1,002 and *B. cereus* BF2, which were all motile, spore‐forming, catalase‐positive and rod‐shaped. All were 0.3% bile salt, 0.4% phenol and pH tolerant. Two‐way ANOVA test P values revealed that *B. cereus* BF2 was statistically significant (*p* < .0014) in bile salt tolerance test. *B. cereus* HKS 1–1 was significant in phenol and pH tolerance at *p* < .0002 and *p* < .0489, respectively. Besides, the test strains showed antibiotic sensitivity and antimicrobial activity to different enteric pathogens. In vivo model referred the test strains as effective in partial allergy reduction at same CFU but at different concentrations with *p* < .0001 among the groups.

**Conclusion:**

The isolated and characterized strains of *B. cereus* showed partial immune‐stimulating potentiality against experimentally induced allergic reaction.

## Graphical abstract

Different *Bacillus cereus* strains were isolated from different buffalo milk samples to analyse their physiological, biochemical and molecular characterization. Their antibiotic sensitivity and antimicrobial activity were also tested to understand whether or not they can be used as therapeutic probiotics. Afterwards, formulation of the test strains at same CFU but at different concentrations was done to apply on the experimental farmed‐ducks to assess their efficacy in suppressing the level of allergy.

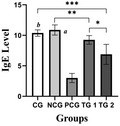

## 1 INTRODUCTION

Hypersensitivity reaction is instigated by allergens which can make touching base with immune systems through inhalation, ingestion, insect bite or skin contact; the phenomenon is then known as allergy. In response to ordinary exposure to allergens (usually proteins), immunoglobulin E (IgE) antibodies are formed and typical symptoms such as asthma, rhino conjunctivitis, eczema/dermatitis etc. may be disclosed (Tang, Chang, & Chen, 2015). For decades, different types and doses of antihistamine drugs have been used to suppress unwanted allergies for human, but things are quite different for animals and birds. Along with the conventional antihistamine treatments, distinguishable natural probiotic feed supplements for chicken, ducks, geese and other birds are suggested. *Bacillus subtilis* is commonly referred as a probiotic immune‐stabilizer against diseases for ducks (Rajput, Li, Li, Jian, & Wang, 2013), while the probiotic potentiality of *Lactobacillus* has already been established for duck immunization (Vasai et al., 2014).

Several studies were found successful using *Bacillus subtilis* in search of immunizing broilers worldwide (Jeong & Kim, 2014). In contrast, the safety of 15 commercial probiotic *B. cereus* isolates has already evaluated in China but the safety remains questionable in terms of mislabeling, toxin production, and transferable antimicrobial resistance (Zhu et al., 2016). *Bacillus cereus* is a Gram‐positive, facultative aerobic bacteria and structurally spore forming rod shaped, having a ubiquitous nature of sources such as soil, decaying organic matter, vegetation, fresh and marine waters, and the invertebrate gut, along with dirt, air, and stools are various sources of the bacterium. It is also found in pasteurized as well as in raw milk of different sources (Bottone, 2010; Gherardi, 2016; Te Giffel, Beumer, Granum, & Rombouts, 1997). Due to the adhesive nature of its endospores, the bacterium spreads to all kinds of food, and thus is associated with food poisoning. Nausea, vomiting, and diarrhea with abdominal cramping can occur within 1 to 6 hours of ingestion of contaminated food (Stenfors Arnesen, Fagerlund, & Granum, 2008).

In recent times, nonpathogenic spores of *Bacillus cereus* have been used as animal feed supplements in diversified ways and options (Mietke, Beer, Schleif, Schabert, & Reissbrodt, 2010). The bacteriocins synthesized by *B. cereus* have found strong therapeutic properties against gut enteric pathogens (Naclerio, Ricca, Sacco, & Felice, 1993), and even the newly identified ‘Cerein’ is considered as the novel bacteriocin (Sebei, Zendo, Boudabous, Nakayama, & Sonomoto, 2007). Though there are seldom research approaches directly mentioning *B. cereus‐*based allergy reduction in domestic animals and birds, *B. cereus* have been found very proactive in suppressing the allergy‐stimulating mites in animal feeds via typical mite‐*B. cereus* symbiosis formation, which newly increased the veterinary and medical importance of *B. cereus* in allergen detection (Erban, Rybanska, Harant, Hortova, & Hubert, 2016).

So, our focus in this study convoluted to the identified strains of *B. cereus* to analyse whether they are functionally probiotic in nature against different types of enteric pathogens and induced hypersensitivity reaction in experimental ducks.

## 2 MATERIALS AND METHODS

### 2.1 Isolation and primary identification

Milk samples of three buffalo breeds namely Nili‐Ravi (Indian), Nili‐Deshi (Indo‐Bangladesh hybrid) and Murrah (Pakistani) were collected from Government Buffalo Farm, Bagerhat, Khulna, Bangladesh. From each sample, primary culture was prepared at dilution up to 10^–11^ times and was inoculated into *Bacillus cereus* selective Polymyxin pyruvate egg‐yolk mannitol–bromothymol blue agar (PEMBA) medium (Holbrook & Anderson, 1980) at 37°C for 24 to 72 hr separately to obtain individual colonies. A total of 29 isolates were prepared from the seventh subculture plate of each sample and three most viable isolates were finally taken for further steps.

### 2.2 Molecular identification of the test strains (Al Azad, Farjana, Mazumder, Abdullah‐Al‐Mamun, & Haque, 2020)

Identification of bacterial strains using 16S rRNA sequencing was approached through RNA extraction, 1.2% Agarose Gel Electrophoresis, isolated RNA amplification with Universal 16S rRNA Specific Primer 8F (AGAGTTTGATCCTGGCTCAG) and 1492R (AAGTCGTAACAAGGTAACC) using Veriti® 99 well Thermal Cycler (Model No. 9902). A single discrete PCR amplicon band of ~1,400 bp was observed for each of the isolates. The PCR amplicons were enzymatically purified and further subjected to Sanger sequencing. Bi‐directional DNA sequencing reaction of PCR amplicon was carried out with 8F and 1401R primers using BDT v3.1 Cycle sequencing kit on ABI 3730xl Genetic Analyzer, in Xcelris Labs Ltd, India. Finally, the FASTA format of the consensus sequences of the bacteria was submitted to NCBI through Gene Bank and the new accession numbers corresponding the bacterial strains were registered and the evolutionary relationships of the strains were analysed by MEGA5.

### 2.3 Characterization for probiotic properties

The morphological (size, shape and motility); biochemical (gram staining and catalase test) and physiological (pH tolerance, bile salt tolerance and phenol tolerance) characterizations were carried out following Barai, Hossain, Rahman, Al Mazid, and Gazi (2018).

### 2.4 Antibiotic sensitivity test

The minimum inhibitory concentration (μg/ml) for the isolates at 50% (MIC_50_) and 90% (MIC_90_) of different selective antibiotics was performed by Epsilometer test (Turnbull et al., 2004).

### 2.5 Antimicrobial activity test

The antimicrobial activity of the isolated strains was observed with the methodology of El‐Banna and Qaddoumi (2016) against clinically pre‐isolated selective enteric pathogens.

### 2.6 In vivo trial against induced allergic reaction

A total of 40 duck eggs were collected from Government Regional Duck Farm (GRDF), Khulna and divided into five groups: Control Group (CG), Negative Control Group (NCG), Positive Control Group (PCG), Treatment Group 1 (TG1) and Treatment Group 2 (TG2). All the groups were provided their respective treatment infused at the second week of hatching. The CG, NCG, PCG, TG1 and TG2 were provided: distilled water, histamine (≥97.0%, Sigma Aldrich), histacin (Jayson Pharmaceuticals Ltd.) with histamine, 50 µl (CFU 30X10^11^) and 100 µl (CFU 30X10^11^) of each test strain, respectively. The pores were sealed with paraffin wax for hatching till the ducklings come out. At the sixth day of post‐hatching, the ducklings of all groups were injected histamine (10 µl of ≥ 97.0%). The onset of allergic reaction started within 6 hr. Finally, they were sacrificed at the seventh day and their blood samples were collected individually for ELISA (Nori Chicken IgE ELISA kit, Catalogue No.GR114113) to evaluate their immunoglobulin E (IgE) level. The ethical guidelines for our in vivo experiment were approved by Animal Ethics Committee, Khulna University, Khulna, Bangladesh (Research Ref. No.: KUAEC‐2017/08/15).

### 2.7 Statistical analysis

All the statistical analysis was performed using Statistical Analysis System (SAS Institute, Cary, NC, USA) for analysis of variance and GraphPad Prism (version 8.0, GraphPad Software, San Diego, CA, USA).

## 3 RESULTS

### 3.1 Isolation and molecular identification

**Figure 1:**
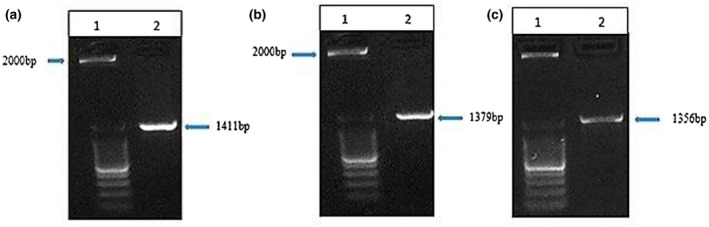
1.2% Agarose gel electrophoresis showing 16S rRNA amplicons of the *Bacillus* where lane 1 indicates 2kb ladder for all and lane 2 indicates 16S rRNA amplicon for *B. cereus* HKS 1–1 (a); *B. cereus* LOCK 1002 (b) and *B. cereus* BF2 (c), respectively

The isolates were *Bacillus cereus* due to their survivability in *B. Cereus* Selective Agar based on highly specific PEMBA medium having Gram‐positive, rod‐shaped, motile, catalase‐positive properties. PCR amplicons of the three isolates showed similar pattern of bands in agarose gel electrophoresis (Figure 1) as all were different strains of the same species*.*

**Figure 2:**
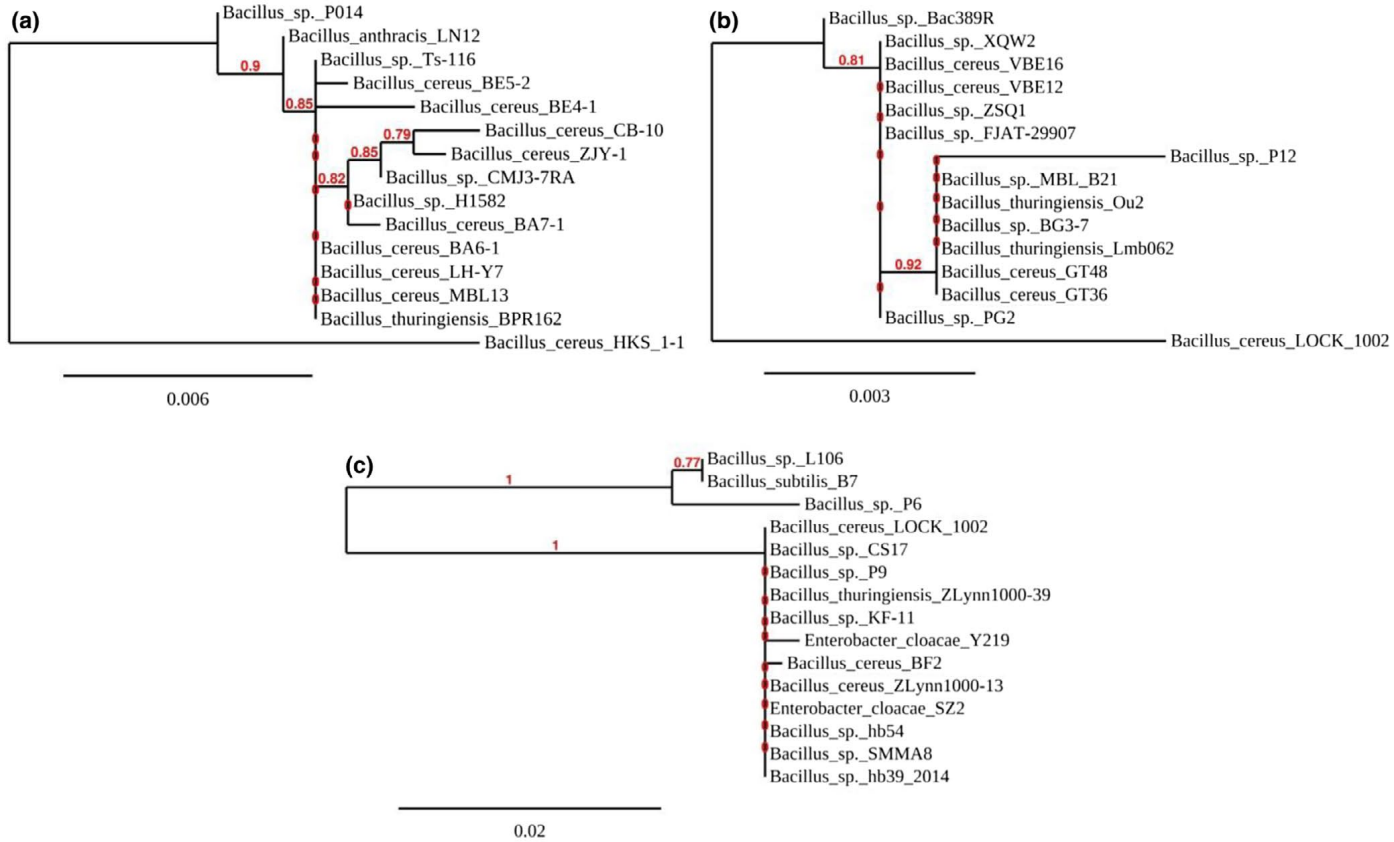
The evolutionary relationship of *B. cereus* HKS 1–1(a); *B. cereus* LOCK 1,002 (b) and *B. cereus* BF2 (c) is illustrated. The accession numbers for *B. cereus* HKS 1–1, *B. cereus* LOCK 1,002 and *B. cereus* BF2 are MH569090.1, MH595555.1 and MH569091.1, respectively. The evolutionary distances were computed using the Kimura 2‐parameter method and are in the units of the number of base substitutions per site. The analysis involved 15 nucleotide sequences for each strain to compare

The banding patterns alike to the figures of the strains of *B. cereus* obtained by [18]. 16S rRNA analysis of Nili‐Ravi, Nili‐Deshi and Murrah isolates identified them as *B. cereus* HKS 1–1, *B. cereus* LOCK 1002 and *B. cereus* BF2, respectively (Figure 2).

### 3.2 Characterizations for the probiotic properties

#### 3.2.1 Biochemical tests of the isolated *B. cereus* strains

Bile salt tolerance test revealed that *B. cereus* HKS 1–1, *B. cereus* LOCK 1002 and *B. cereus* BF2 were tolerant at 0.3% concentration while, *B. cereus* BF2 (*p* < .0014) were statistically significant (Table 1). All of the strains were sensitive to grow at low pH (acidic conditions) but their survivability increased with the increase of pH level (Table 1), where *B. cereus* HKS 1–1 (*p* < .0489) was statistically significant and all of the test strains were found tolerant to phenol at 0.4% (Table 1), when *B. cereus* HKS 1–1 was highly significant (*p* < .0002) among the test strains.

**Table 1:** Physiological characterization of the isolated *B. cereus* HKS 1–1 (†), *B. cereus* LOCK 1,002 (‡) and *B. cereus* BF2 (§) is mentioned at 24 hr of incubation, with all significant (a) Two‐way ANOVA test *p* values (in scale of significance *p* < .05). The *p* values are .0014* (§), .0489** (†) and .0002*** (†) (which are all significant values for bile salt tolerance, pH tolerance and phenol tolerance test, respectively). In all cases, ‘‡’ was statistically insignificant

pH **	OD_650_			Bile Salt (%)*	OD_650_			Phenol (%) ***	OD_516_		
	24 hr				24 hr				24 hr		
	†a	‡	§		†	‡	§a		†a	‡	§
2	0.43	0.486	0.507	0.1	0.144	0.152	0.149	0.1	0.625	0.744	0.624
3	0.334	0.35	0.33	0.2	0.156	0.157	0.155	0.2	0.619	1.355	0.648
4	0.356	0.35	0.35	0.3	0.164	0.164	0.155	0.3	0.626	1.825	0.63
								0.4	0.637	2.205	0.626

#### 3.2.2 Antibiotic sensitivity test

The strains of *B. cereus* were found susceptible to different antibiotics (Table 2) in the Epsilometer test (E‐test) method, which was referred as an authentic way of high‐throughput sensitivity detection by Turnbull et al. (2004). All the test strains of *B. cereus* were highly sensitive to erythromycin as the range was 0.066 µg/ml–0.093 µg/ml to result MIC_50_ when MIC_90_ resulted in the range of 1.53 µg/ml–1.81 µg/ml. In contrast, lowest sensitivity was observed for streptomycin with the range of 34–36 µg/ml and 48–58 µg/ml to obtain MIC_50_ and MIC_90_, respectively (Table 2).

**Table 2:** MIC for the most effective antibiotics of the antibiotic sensitivity test of the *B. cereus* strains has been provided where Epsilometer test ^a^ (E‐test) for MIC was used. MIC_90_ and MIC_50_ imply minimum inhibitory concentration for 90% and 50% inhibition of the test microorganism, respectively. Amphotericin B ^b^ (AmB) is a polyene antibiotic frequently applied in the treatment of fungal infections. The unit was used in µg/ml

Antibiotics	Method a	MIC (µg/ml) for the *Bacillus cereus* strains								
		*B. cereus* HKS 1–1			*B. cereus* LOCK 1002			*B. cereus* BF2		
		Range	MIC_50_ †	MIC_90_ ‡	Range	MIC_50_ †	MIC_90_ ‡	Range	MIC_50_ †	MIC_90_ ‡
Tetracycline	E‐test	0.08–34	1.5	7	0.083–30	1	6.25	0.05–32	1	7
Streptomycin	E‐test	16–64	34	48	16–60	35	54	16–62	36	58
Penicillin G	E‐test	0.012–>32	30	32	0.015–>32	32	32	0.014–>36	32	36
Vancomycin	E‐test	1–16	3	6	1–16	4	8	1–16	4	8
Cefotaxime	E‐test	0.1–>30	>27	>30	0.1–>28	>21	≥28	0.1–>31	≥23	>31
Gentamicin	E‐test	0.099–≥0.84	0.46	≥0.84	0.094–≥0.79	0.51	≥0.79	0.091–≥0.73	0.48	≥0.73
Erythromycin	E‐test	0.035–3.05	0.081	1.63	0.035–3.05	0.093	1.81	0.032–3.05	0.066	1.53
Amoxicillin	E‐test	1.6–16	8	16	1.6–16	8	16	1.6–17	8.4	17
^b^Amphotericin B	E‐test	0.03–1	0.575	0.875	0.03–1	0.525	0.775	0.03–1	0.8	1

^†^ Minimum inhibitory concentration for 50% inhibition (MIC_50_)

^‡^ 90% inhibition (MIC_90_)

#### 3.2.3 Antimicrobial activity test

Antimicrobial activity of the isolated *B. cereus* strains was phenomenal with a wide range of inhibition patterns (Table 3)**.** *B. cereus* HKS 1–1 showed its effectiveness against *Pseudomonas aeruginosa* at around 10 mm diameter of inhibition. On the other hand, *B. cereus* LOCK 1002 and *B. cereus* BF2 showed partial antagonism against *Salmonella paratyphi* and *Staphylococcus aureus*. Interestingly, *B. cereus* BF2 failed to restrict *E. coli* growth in culture condition. Among the test strains, *B. cereus* HKS 1–1 had maximum and *B. cereus* BF2 showed minimum antagonistic traits against the selective pathogens (Table 3)**.**

**Table 3:** Antimicrobial activities of the test strains of the isolated *B. cereus* strains against selective enteric pathogens are presented. Range of inhibition: +, diameter of inhibition zone 2–5 mm; ++, 6–9 mm; +++, ≥10 mm; ‐, resistance to the test strain/s

Test	Pathogens							
strains	Zones of inhibition (mm)							
	*E. coli* ^b^	*V. cholerae*	*S. typhi*	*S. paratyphi*	*Micrococcus*	*P. aeruginosa* ^a^	*B. megaterium*	*S. aureus*
*B. cereus* HKS 1–1	++	++	++	++	++	+++	++	+
*B. cereus* LOCK 1,002	++	++	++	+	++	++	++	+
*B. cereus* BF2	‐	+	+	+	+	+	+	+

^a^ *P. aeruginosa* showed maximum

^b^ *Escherichia coli* had minimum sensitivity

### 3.3 In vivo sampling and data analysis

**Figure 3:**
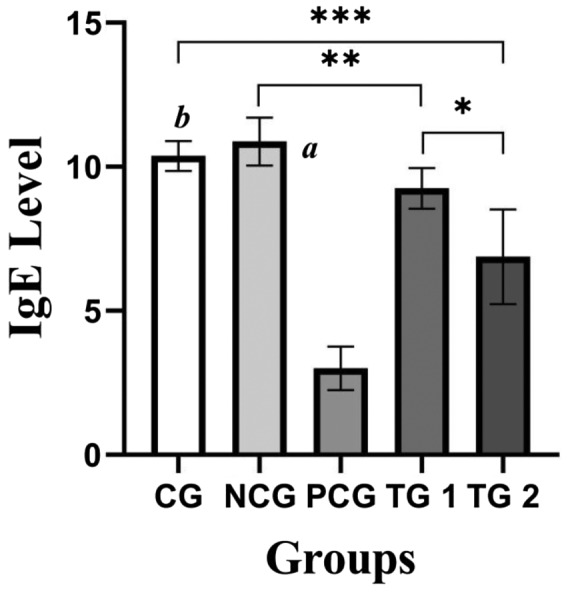
Immunoglobulin E (IgE) level comparison among the collected blood samples from different in vivo groups, which shows the two‐way ANOVA*** among the groups presented *p* < .0001 which is highly significant. *p* value from *t* test is <.0067* and <.0034** between the Treatment Groups 1 and 2 and between the Negative Control Group and Treatment Group 1, respectively; except CG, *p* < .0001*^a^* when others are compared with the NCG; *p* < .0001***^b^*** when TG 1 and TG 2 are compared with CG, except the NCG and PCG (which are all significant values, in scale *p* < .05)

In this research, all the experimental ducks showed differential anti‐allergic response considering their in ovo belonging groups and their corresponding treatment agents during the post‐hatch (in vivo) period of histamine‐induced allergic reaction. TG2 executed slightly more immune stimulation against allergy than TG1 (Figure 3), which mentioned the dose effects of the combined testing of *B. cereus* strains.

The result of PCG was the most significant in boosting anti‐allergic action as histacin was used for standardization. In contrast, NCG was the worst to response against histamine with a high pick of IgE level (Figure 3).

## 4 DISCUSSION

Bacterial growth on PEMBA medium preliminary identified them as *Bacillus cereus* due to their survivability which was previously reported by Holbrook and Anderson (1980) and properties like Gram‐positive, rod‐shaped, coagulase‐positive, catalase‐positive and motility which directly matched with the findings of Bottone (2010). The phylogenetic trees have covered 15 different bacterial species for each test strain with reference to Figure 2. Identification of the target strains of *B. cereus* was done on the basis of their high similarities to the reference strains in the Gene Bank. Eight different strains of *B. cereus* were found with their maximum similarities with *B. cereus* HKS 1–1, including *B. cereus* BA6‐1, *B. cereus* BE5‐2, *B. cereus* BE4‐1, *B. cereus* BA7‐1, *B. cereus* ZJY‐1, *B. cereus* LHY‐7, *B. cereus* CB‐10 and *B. cereus* MBL13 (Figure 2a). *B. cereus* LOCK 1002 was found highly similar to *B. cereus* GT36 according to their query coverage and maximum score (Figure 2b), while *B. cereus* BF2 had significant evolutionary pattern with *B. subtilis* B7, *B. cereus* LOCK 1002 and *B. cereus* ZLynn1000‐13. Besides, *Enterobacter cloacae* SZ2 had evolutionary relationship with *B. cereus* BF2 outside the *Bacillus* members (Figure 2c).

The evolutionary history was inferred using the Neighbour‐Joining method as referred by Saitou and Nei (1987). Following Felsenstein (1985), the bootstrap consensus tree inferred from 1000 replicates was taken to represent the evolutionary history of the taxa analysed. Branches corresponding to partitions reproduced in less than 50% bootstrap replicates were collapsed.

The test strains were tolerant to 0.3% even after 24 hr of incubation and *B. cereus* BF2 was the most significant survival among the others (Table 1). The same bile salt tolerance capabilities have already been reported to happen for other *B. cereus* strains like *B. cereus* BF2 by Kristoffersen et al. (2007) and even for some established probiotics like *Lactobacillus* spp. and *Bifidobacterium* spp. as explained by Barai et al. (2018) and Abdullah‐Al‐Mamun et al. (2016). *B. cereus* survivability increases with the promotion of basic nature of growth media unlike *Lactobacillus* and few other established probiotics. Survivability at pH level 5.0 and below is completely stagnated in our research, in contrast, *Lactobacillus* spp. can easily survive at pH 3.0, mentioned by Barai et al. (2018). The result also reflects the findings of Desriac et al. (2013) about *B.* cereus acidophilic behaviours. Besides, the phenol tolerance properties of the test strains of our research at different concentrations suit well to the biochemical properties of *B. cereus* (Table 1) according to Singh, Singh, and Chandra (2009) who stated that few strains of *B. cereus* remain viable in a wide range of concentrations and biodegradation of phenol in batch culture by *B. cereus* pure culture and/or mixed culture with *Paenibacillus* spp. Even bioremediation of petroleum wastewater by hyper‐phenol tolerant *B. cereus* has also reported by Banerjee and Ghoshal (2017).

The strains of *B. cereus* were found susceptible to different antibiotics (Table 2) in the Epsilometer test (E‐test) method, which was referred as an authentic way of high‐throughput sensitivity detection by Turnbull et al. (2004). Besides, the result of our research directly reflects the response of *Bacillus* spp. to different antibiotics similar to the approach of Coonrod, Leadley, and Eickhoff (1971).

In our present study, all of the three *B. cereus* strains showed effective antagonistic effects to the selective enteric pathogens such as *E. coli* (Karthikeyan & Sahayarayan, 2017; Kumar, Thippeswamy, Kuppust, Naveenkumar, & Shivakumar, 2015); *V. cholera, S. aureus* (Gupta, Gauri, & Shrivastava, 2013); *S. typhi, S. paratyphi; P. aeruginosa* (Kumar et al., 2015); *Micrococcus* and *B. megaterium*. *B. cereus* HKS 1–1 showed most positive antimicrobial activity to *P. aeruginosa*, while *B. cereus* LOCK 1002 and *B. cereus* BF2 showed most against *E. coli* and *S. paratyphi* (Table 3), respectively. *B. cereus* BF2 was found insensitive to *E. coli*. Comparing among the three strains, *B. cereus* HKS 1–1 was highly significant in overall antagonism to the enteric pathogens. *B. cereus* encodes bacteriocin‐like cerein 7, which has been found very effective against different pathogens as mentioned by Naclerio et al. (1993) and Oscáriz, Lasa, and Pisabarro (1999).

The two‐way ANOVA report of IgE testing among the in vivo groups was highly significant (*p* < .0001) and even the value remained same when all groups were compared with the NCG, except CG. The P value from *t* test is <0.0067 and < 0.0034 between the TG1 and TG2 and between the NCG and TG1, respectively (Figure 3).

The findings of the in vivo IgE test can be considerably innovative as there are seldom established research papers available that directly reflect the anti‐allergic potentialities of *B. cereus* strains. Besides, several established works provide the anti‐allergic activity of *Bacillus* spp. Vogel et al. (2008) found anti‐allergy immunity‐inducing properties in animals by *Bacillus licheniformis*. On the other hand, *Bacillus subtilis* contains positive anti‐allergic potentiality in humans when the spores were used as adjunctive treatments against food allergy as it was clinically proved by Ciprandi, Scordamaglia, Ruffoni, Pizzorno, and Canonica (1986). In addition, Swartzendruber and Knight (2015) reported that *B. subtilis* exopolysaccharide (EPS) can effectively suppress mast cell‐dependent degranulation and anaphylaxis (severe systemic allergic reaction) and in that sense *B. subtilis* was considered as probiotic.

Therefore, the recent findings of our research can be an evidence that *B. cereus* HKS 1–1, *B. cereus* LOCK 1002 and *B. cereus* BF2 have partial anti‐allergic effects in vivo*,* when they are formulated properly as combined doses for each experimental organism. The three newly identified and characterized *B. cereus* strains can be used as biocompatible and nutritious supplements with either basal diets or therapeutics for ducks as alternative preventives to suppress duck allergy allowing chemical hazard‐free immunization and reproduction.

## CONFLICT OF INTEREST

The authors have no competing interest.

## AUTHOR CONTRIBUTION

**Salauddin Al Azad:** Conceptualization; Data curation; Formal analysis; Investigation; Methodology; Software; Visualization; Writing‐original draft; Writing‐review & editing. **Khondoker Moazzem Hossain:** Funding acquisition; Project administration; Supervision; Validation. **Sana Mohammad Mahbubur Rahman:** Project administration; Supervision; Validation. **Mohammad Faysal Al Mazid:** Data curation; Software; Visualization; Writing‐review & editing. **Pallob Barai:** Formal analysis; Software; Visualization; Writing‐review & editing. **Mohammad Shamim Gazi:** Formal analysis; Visualization.

## ETHICAL STATEMENTS FOR ANIMAL RESEARCH

The care and handling were according to the ethical guidelines approved by Animal Ethics Committee, Khulna University, Khulna, Bangladesh (Research Ref. No.: KUAEC‐2017/08/15), which agree with the EU Directive 2010 for animal experiments.
